# *De novo* genetic variation revealed in somatic sectors of single Arabidopsis plants

**DOI:** 10.12688/f1000research.2-5.v2

**Published:** 2013-07-30

**Authors:** Marianne T Hopkins, Aaron M Khalid, Pei-Chun Chang, Karen C Vanderhoek, Dulcie Lai, Meghan D Doerr, Susan J Lolle

**Affiliations:** 1 Department of Biology, University of Waterloo, Waterloo, N2L 3G1, Canada; 2 Department of Pathology and Molecular Medicine, Queen's University, Kingston, K7L 3N6, Canada

**Keywords:** Arabidopsis, genome instability, genetic heterogeneity, mosaicism, inbreeding

## Abstract

Concern over the tremendous loss of genetic diversity among many of our most important crops has prompted major efforts to preserve seed stocks derived from cultivated species and their wild relatives. *Arabidopsis thaliana* propagates mainly by self-fertilizing, and therefore, like many crop plants, theoretically has a limited potential for producing genetically diverse offspring. Despite this, inbreeding has persisted in Arabidopsis for over a million years suggesting that some underlying adaptive mechanism buffers the deleterious consequences of this reproductive strategy. Using presence-absence molecular markers we demonstrate that single Arabidopsis plants can have multiple genotypes. Sequence analyses reveal single nucleotide changes, loss of sequences and, surprisingly, acquisition of unique genomic insertions. Estimates based on quantitative analyses suggest that these genetically discordant sectors are very small but can have a complex genetic makeup. In ruling out more trivial explanations for these data, our findings raise the possibility that intrinsic drivers of genetic variation are responsible for the targeted sequence changes we detect. Given the evolutionary advantage afforded to populations with greater genetic diversity, we hypothesize that organisms that primarily self-fertilize or propagate clonally counteract the genetic cost of such reproductive strategies by leveraging a cryptic reserve of extra-genomic information.

## Introduction

Plants live in ever changing environments and must adapt using strategies that fundamentally differ from those employed by animals. Developmental plasticity is at the core of those strategies allowing plants to modify their growth and the organs they produce in response to different environmental signals. This type of open-ended modular development enhances survival because damaged or diseased units can readily be discarded without compromising viability. Furthermore, because plants are constrained to sessile life styles, a modular growth habit affords greater versatility allowing phenotypic and genetic variation between modules to be used to the plant’s advantage, aiding adaption to pathogen life cycles ^1^ or to longer-term environmental perturbations such as climate change. As a consequence of this profound developmental versatility, even individuals composed of cell populations derived from different plant species are viable and can coordinate the growth and development of chimeric organs ^2^. In an elegant paper published in 1981, Whitham and Slobodchikoff ^3^ proposed that mosaicism offers a unique adaptive advantage for plants by allowing introduction of genetic variants into the gene pool either through vegetative propagation or through sexual reproduction. They further propose that mutations arising somatically have a greater probability of being incorporated into the gene pool than mutations that arise in the gametes ^3^ precisely because germ line cells are derived from somatic tissues that arise late in the developmental history of the plant ^4, 5^.

The relatively frequent occurrence of mosaics among various plant species has been extensively utilized in the development of novel ornamentals and for the selection and maintenance of desirable traits in many cultivated crops. Any desirable cultivars that have arisen in this manner have been maintained through vegetative propagation and, to date, are responsible for a significant fraction of agriculturally important perennial plants. On the other hand, desirable traits in many important annual crops, such as rice, soybean, maize and wheat, have been introduced through classical genetic manipulations using directed breeding strategies. Once generated, annuals with good agronomic performance are usually maintained by inbreeding.

In recent years, concern has grown over the presumed loss of genetic diversity resulting from the application of modern horticultural and breeding practices. Therefore, the benefit of excellent performance may come with a significant cost ^6, 7^. However, recent and surprising results suggest that even highly inbred species harbor unanticipated sources of intrinsic genetic variation. For example, highly inbred soybean cultivars have been shown to manifest significant phenotypic and genetic variation in the absence of sexual manipulation ^8– 10^. Such high intrinsic genetic variation has also been demonstrated for a number of other crop plants ^11^.

In the natural world, inbreeding occurs in many highly successful flowering plant species including wild relatives of *Arabidopsis thaliana* ^12^. Therefore, in nature species that are highly inbred have persisted despite their predicted reduction in genetic diversity. Why would such inbreeding strategies be successful and what are the implications from an adaptive perspective? One possibility put forward by Barrett ^13^ is that such populations are very successful in their particular niches and benefit from producing large numbers of genetically identical offspring. Nevertheless, selection should favor plant species that can co-evolve on time scales reflecting particular environmental challenges such as fluctuations and variations in pathogen populations. In keeping with this view, it has been shown that sequence variation in 20 diverse strains of Arabidopsis is highly non-random. In gene families mediating biotic interactions, such as those implicated in pathogen defense, variation far exceeds that seen in families involved in basic biological processes ^14^.

The underlying mechanisms driving phenotypic variation in highly inbred lines, whether domesticated or wild, have often been inferred and have had limited experimental verification. Nevertheless, relatively simple molecular approaches have provided insight into some of the genomic events coinciding with visible changes in phenotype. In flax, for example, molecular assays have demonstrated that heritable phenotypic changes induced by environmental shifts are accompanied by reproducible changes in genomic DNA including changes in total DNA content, non-random changes in DNA sequences or sequence rearrangements ^15– 18^. In soybean, reproducible non-random DNA sequence changes induced by *in vitro* culturing of root explants have also been demonstrated using restriction fragment length polymorphic markers ^19^. Genomic changes manifesting similar hallmarks of biased sequence alterations have also been described for banana ^20^ and in rice hybrids ^21^.

In the work described by Roth *et al.* ^19^ soybean root explants were shown to repeatedly give rise to particular alleles that were absent in the donor plants but had previously been found and characterized in other varieties of cultivated soybean. To account for the appearance of these particular allelic variants the authors proposed that these organisms had evolved "internal generators of genetic variation" that mediated genome changes through some type of recombination process. In 2005, Lolle and colleagues ^22^ described a genome-wide phenomenon in Arabidopsis *hothead* ( *hth*) mutants that was very reminiscent of that described by Roth *et al*. ^19^. Based on the nature and genome-wide locations of the sequence changes detected, it was proposed that a template-directed process mediated these changes and that these cryptic but stable extra-genomic templates themselves had persisted since at least the grandparental generation. Not surprisingly, this proposal met with considerable skepticism and numerous alternative explanations for these data have since been published ^23– 28^.

In this study we have employed presence-absence molecular markers to test for non-Mendelian inheritance and found that Arabidopsis plants can inherit novel insertion sequences that were absent in their immediate parents. Furthermore, we show that discordant DNA-based marker profiles can be found between tissues isolated from different parts of an individual plant. These experiments demonstrate that individual plants spontaneously produce somatic sectors and are genetic mosaics. Since genetic variation can occur in the same plant in the absence of sexual reproduction, we propose that these novel insertion sequences must originate from cryptic reserves intrinsic to the host plant itself. The data presented support the original contention that a previously unknown template-directed mechanism exists ^22^ and raise the encouraging possibility that other inbreeding species, including crop plants, may also harbor a cryptic reserve of genetic variation.

## Methods

### Plant material and growth conditions

**Figure 1.**
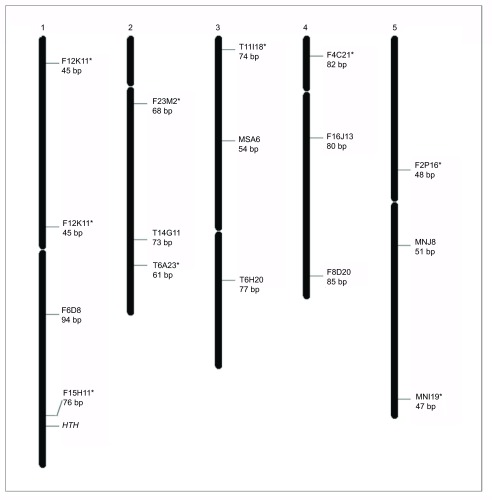
Haploid representation of the 5 Arabidopsis chromosomes indicating the relative locations of the 16 insertion-deletion polymorphic markers used in this study. Nine of the markers are intergenic (*). Marker names reflect clone designations. The size of the insertion sequence is indicated in base pairs (bp). The relative location of *HOTHEAD* ( *HTH*) is shown at the bottom of chromosome 1.

All genetic stocks of *Arabidopsis thaliana* used for these experiments have been described previously ^29^. Arabidopsis seeds derived from these stocks were sown onto moistened potting mix (1:1 mixture of LC1:LG3 Sungro Sunshine potting mixes, Sungro Horticulture, Seba Beach, AB) and stratified at 4°C for 2–5 days. Plants were maintained in growth chambers (Econoair AC60, Ecological Chambers Inc., Winnipeg, MB; GC8-VH/GCB-B, Environmental Growth Chambers, Chagrin Falls, Ohio; Conviron PGW36/E15, Controlled Environments Ltd., Winnipeg, MB) and illuminated with a mixture of incandescent and fluorescent lights (140–170μmol m ^-2^ sec ^-1^ at pot level) with a 24-hour photoperiod. Growth chambers were maintained at 20 ± 4°C at 40–60% relative humidity. Plants were grown in flats or in 3- or 6-inch pots and watered as needed. Seeds used for seedling root-shoot comparison were surface sterilized using bleach and plated on agar medium containing half strength MS basal salts (Sigma, St. Louis, USA). Seedlings were harvested approximately 5 days post-germination. Hybrid lines were generated between wild type Lansberg *erecta* plants or homozygous *hth* mutant lines in the Landsberg *erecta* background and Columbia accessions by manual pollination. All crosses were done reciprocally. F2 seed was obtained from self-fertilized F1 plants. Individual F2 plants were reared in plastic tubes (Johnston Industrial Plastics, Ontario, Canada) and F3 seed collected from each F2 plant individually. Tissue samples were collected from individual F2 and F3 plants, and genotypic profiles were determined using insertion-deletion polymorphic molecular markers (see Figure 1).

### Out-crossing experiments

Experimental set ups were replicated twice and the net out-crossing frequencies determined. Herbicide-resistant transgenic Arabidopsis pollen donors previously transformed with the pCB302 mini binary vector only ^30^ and mutant test plants were grown in a 1:1 ratio and arranged in randomized positions ( www.random.org). Out-crossing frequencies were also compared to plants under the same conditions but reared within plastic tubes. Progeny were sprayed with glufosinate (40 micrograms ml ^-1^ active ingredient: WipeOut, Nu-Gro IP Inc., Ontario) to test for herbicide resistance and resistant plants tested for segregation of *hth* mutant progeny plants.

### DNA extraction and molecular genotyping

For DNA extraction, rosette or cauline leaf tissue was collected and DNA extracted according to the method of Edwards *et al* ^31^. Samples not processed immediately were stored at -20°C. To distinguish the mutant *hth-4* allele from the wild type, genomic DNA was amplified using oligonucleotide primers immediately flanking the *hth-4* point mutation (5'GAAGCTGGTGAAGGAGTCGT-3', 5'-CTCCGCCGCGGTGTGTC-3'). The resulting 205 base pair (bp) PCR product was then digested with *Sal*I restriction endonuclease (New England Biolabs) and endonuclease treated PCR products size separated by agarose gel electrophoresis. Sixteen sets of DNA oligonucleotide primers were designed to amplify approximately 150–300bp genomic regions by polymerase chain reactions (PCR), each containing one 45–94bp marker which is present in the Columbia but absent in the Landsberg accession ( Table 1). PCR amplicon products were size separated by agarose gel electrophoresis.

**Table 1.** List of primer pairs used for PCR-based molecular genotyping. Expected amplicon product sizes for the Columbia and Landsberg accessions are shown in adjacent columns.

Insertion-deletion marker	Primer pairs	Columbia product size in base pairs	Landsberg product size in base pairs
F12K11	ccatatcttggagttggcaga tgtcttcaggaacacaacca	166	121
F5J5	tgaagatttcgtggaagcaa ctcatggatgcctaataccg	275	200
F6D8	ctccgtcttccagagtttga ttcgggtgattagtacggaaa	211	107
F15H11	atttgcggctgaaagacaag tgagtgtgtcatgagtgtttgttt	229	153
F23M2	taaagttgttggccgaggag tcggagatacccgagctaaa	231	163
T14G11	cctatgtgtcaagagagatttcca tttgttccatttataagcgtttctc	286	213
T6A23	aacaccaagtcaactgtttttgtt tcaaaataaacacccccaact	241	180
T11I18	ccccaattcgaaatgtaagg cgctccttgacagttttcct	203	129
MSA6	ctggggtgttctcacaggat cgttggaggtggtcttaggt	199	145
T6H20	tgcattggtttctctgcttg gggaaacctccatactcgaa	231	154
F4C21	tggttagggttctggtcagg agtggctcatcgttcgagat	195	113
F16J13	gaagcatgttttgtgtatcttgc ccgcatctccacatttcatt	224	144
F8D20	caccagacggtgatgaagag cattcgcgcatttattgttg	202	117
F2P16	aaaatggtttaccacatggaca tcccaaatcaattcaaggaaa	223	175
MNJ8	catggatcaaagatgatctcca ttcgcttttcgtgtttctga	184	133
MGI19	tgcacatgacttcaacagaaaa atgtgggtgggtgttgattt	203	156

### Isolation, cloning and sequencing of PCR products

Portions of genomic DNA were PCR amplified and sequenced directly or products cloned into standard pGEM TA vectors (Promega). Amplified or cloned PCR products were sequenced at the Centre for Applied Genomics ( http://www.tcag.ca/, Toronto, Ontario). Sequence alignments were generated using CLC Sequence Viewer 6.4 software (CLC bioA/S; www.clcbio.com).

### qPCR methods

Quantitative PCR was performed on a Bio-Rad Real-Time thermal cycler CFX96 attached to a computer running CFX Manager. SsoFast EvaGreen Supermix (Bio-Rad) was used according to manufacturer’s instructions. A series of primers either flanking or internal to the insertion sequences were used to generate control and experimental amplicons. The positive control was a PCR product amplified from the Columbia accession, spanning the indel sequence of interest by ~700–900bp. The positive control was gel purified and used to generate a standard curve for conversion of C(t) value to copy number of the insertion sequence and the external reference sequence. External reference primers immediately flanked the indel markers. Insertion sequences were detected using one external reference primer paired with a primer homologous to sequences within the insertion itself. Primer sequences and amplicon product sizes are listed in Table 2. The colors indicated in the first column (insertion-deletion marker) correspond to the colors used for the qPCR-generated bar graphs.

**Table 2.** List of primer sets used for qPCR analyses. Primer positions, left and right primer sequences and expected amplicon sizes are indicated for each marker. Colors correspond to those used in Figure 5B and Figure 6B.

Insertion-deletion marker	Primer position	Left primer sequence	Right primer sequence	Product size in base pairs
**F6D8**	Positive control	ctgaccagcaaattctcaagg	tgagcaggtgaaacagatgg	766
	External reference	aagtttaaaacgaaaactttataaaatacc	tttcgtgttcgtggttttca	214
	Within insertion	aaacaagtgcatgttgcg	tttcgtgttcgtggttttca	266
**F15H11**	Positive control	ctccactaactcccgttattcc	gaacaatcgggccacatatag	701
	External reference	tttcgtcacttttcaaaactaac	gtgtgtgtgtgtgtgtgtgctc	151
	Within insertion	tgatgattttggattgaacgtc	gtgtgtgtgtgtgtgtgtgctc	201
**T14G11**	Positive control	gagttgtgttccagggccta	tttgttgtgcgaattcattg	897
	External reference	cacaaaaattaaggaataataaatgttctc	tttgttccatttataagcgtttctc	143
	Within insertion	ttgtcccattttatttgatgtttg	tttgttccatttataagcgtttctc	176
**T6H20**	Positive control	tttcctgtttgggatctgag	tcaggagatagtccaccatgc	839
	External reference	tgggcttaccctgttcatggag	gcagagaaaccaatgcattttca	151
	Within insertion	tgggcttaccctgttcatggag	ccagaaaccgagtctctaagatttca	259
**MGI19**	Positive control	atatgcttgtcagtgagggaag	gaattcgacaggagcgtgaag	800
	External reference	gaacaatttgtggaaaaatggaa	cctagtttcatgtgcatatatgtc	181
	Within insertion	gaacaatttgtggaaaaatggaa	tgacatgtactcaccgcaatg	212

## Results

### Mutant *hth* plants are susceptible to higher rates of out-crossing

Homozygous *hth* mutant Arabidopsis plants were previously shown to give rise to wild type (wt) progeny at relatively high frequencies ^22, 29^. Although an intrinsic mechanism was proposed ^22^, cross-pollination with neighboring plants was subsequently put forward as the more likely explanation for the appearance of these wt revertant offspring ^26, 27^. To test the susceptibility of *hth* plants to out-crossing under our growth conditions, experiments were conducted using a pollen donor harboring a dominant gene conferring resistance to the herbicide glufosinate. Herbicide-resistant transgenic lines were grown together with *hth* and *eceriferum-10 (cer-10)* ^32^ floral fusion mutants and wt Landsberg plants. These analyses confirmed that the majority of *hth* mutant plants did not cross-pollinate. However, when cross-pollination was detected, frequencies varied considerably between individual *hth* mutant plants. Mutants with floral fusion phenotypes were predisposed to higher pollen capture than wild type plants (0.02–0.43% for *hth-4, 8 and 10* mutants, 0.89% for *cer10* mutants, 0.01% for wt plants). In addition, factors such as donor-recipient proximity, the severity of the floral fusion phenotype, growth chamber airflow patterns and plant handling influenced the propensity to cross-pollinate. Nevertheless, growing *hth* mutant F2 plants in the complete absence of *HTH* pollen donors did not eliminate wt progeny from F3 progeny pools and, on average, 1.53% of F3 progeny were phenotypically wt for *HTH* despite being derived from self-fertilized homozygous F2 *hth* mutant parent plants (2/133 *hth-4*, 2/131 *hth-8* and 2/127 *hth-10* gave rise to wt F3 progeny). Under our laboratory conditions, out-crossing could not be completely eliminated within *hth* mutant populations if mutants were grown together with wt plants, even if every *hth* mutant plant was shielded in transparent plastic tubes.

**Figure 2.**
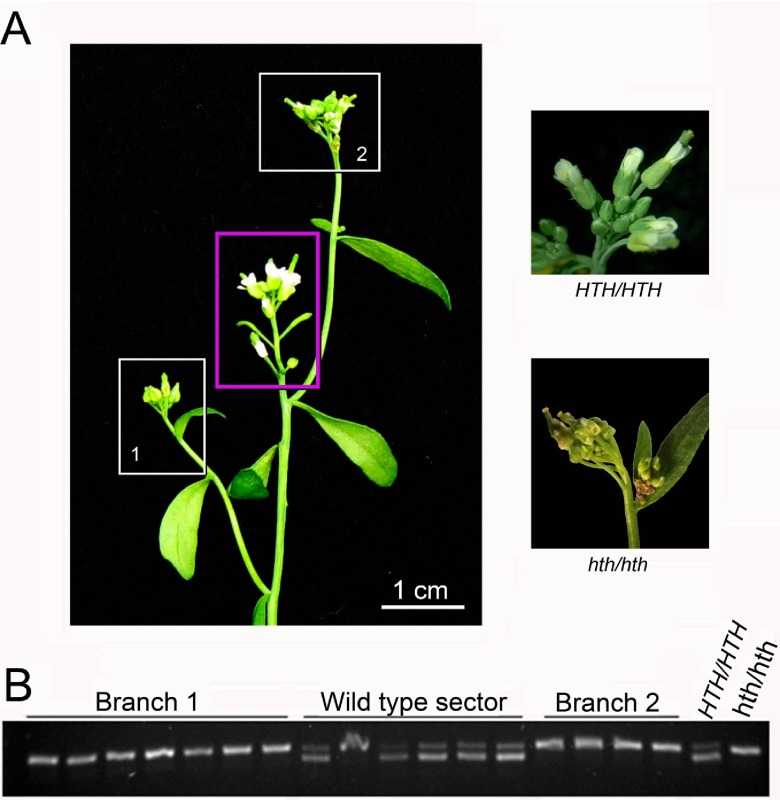
Molecular analysis of a mutant *hth-4* plant showing a large wt sector. ( **A**) Two mutant branches (white boxes) flank a phenotypically wt flower branch (magenta box). Examples of normal wt ( *HTH/HTH*) and mutant ( *hth/hth*) flowers are shown on the right. ( **B**) DNA was extracted from tissue samples and allele-specific PCR-based molecular markers used to determine genotype. The wt branch scored as heterozygous ( *hth-4/HTH-4*), while mutant branches scored as homozygous for the *hth-4* allele.

While conducting segregation analyses and scoring offspring for herbicide resistance, a single *hth* mutant plant with a large phenotypically wt floral sector was identified ( Figure 2). Sampling of shoot tissues confirmed that phenotype corresponded to genotype and that both mutant *hth-4* and wt *HTH* alleles could be detected in tissue derived from this large wt sector ( Figure 2B).

The identification of this sectored individual provided the first phenotypic evidence that *hth* plants were capable of producing somatic sectors. This finding suggested that perhaps some of the wt revertants originally found among *hth* mutant progeny might have arisen from genetically heterozygous sectors on the parent plant ^22^. Since well over 300,000 mutant plants were screened in the course of our out-crossing experiments and only one plant with a very large phenotypically wt sector found such as that shown in Figure 2B, we reasoned that if sectoring does occur, the vast majority of sectors would be too small to result in a visible phenotype. This possibility prompted us to test whether novel genotypes could be detected in tissue samples obtained from single *hth* plants.

### Single plants can have multiple genotypes

For these experiments we chose to focus exclusively on molecular markers consisting of genomic DNA sequence tracts between 45–94 nucleotides in length that are either present or absent in the Columbia and Landsberg Arabidopsis accessions (insertion-deletion polymorphic markers or indels; Figure 1). In choosing to use indel markers we reasoned that deletions would be recalcitrant to enzyme repair or modification and therefore would help differentiate between enzyme-based mechanisms such as the one put forth by Comai and Cartwright ^24^ and a template-directed mechanism like the one previously proposed ^22^. Hybrid F1 plants were constructed between Columbia and Landsberg accessions by manual cross-pollination, F1 plants allowed to self-seed and F2 and F3 descendants used as experimental material. The Columbia accession was always wt for *HTH* while *hth* mutant alleles, when introduced in hybrid lines, originated from the Landsberg genetic background. For all of the indel markers used in this study, Columbia is homozygous for the insertion.

Initially, F3 seed progeny derived from hybrid F2 parent lines with known indel marker profiles were screened to test whether or not these markers were stable. All F2 parent plants were reared in plastic tubes to minimize outcrossing. When marker profiles were compared between *hth-4* parent plants and their F3 adult offspring, 2.16% [6/277] deviated from the expected profile. This frequency is approximately 5 times higher than baseline rates (0.02–0.43%) seen in outcrossing experiments described above. When F3 progeny were assayed as seedlings, similar frequencies were seen, with 2.5% [15/600] of the F3 seedlings showing discordant marker profiles. Altogether 600 seedlings were tested using a total of 30 seedlings per F2 plant (eleven *hth-4,* five *hth-7,* two *hth-8* and two *hth-10* F2 plants). Of the 15 F3 seedlings that tested positive for at least one non-parental marker, 7 had acquired insertions.

**Figure 3.**
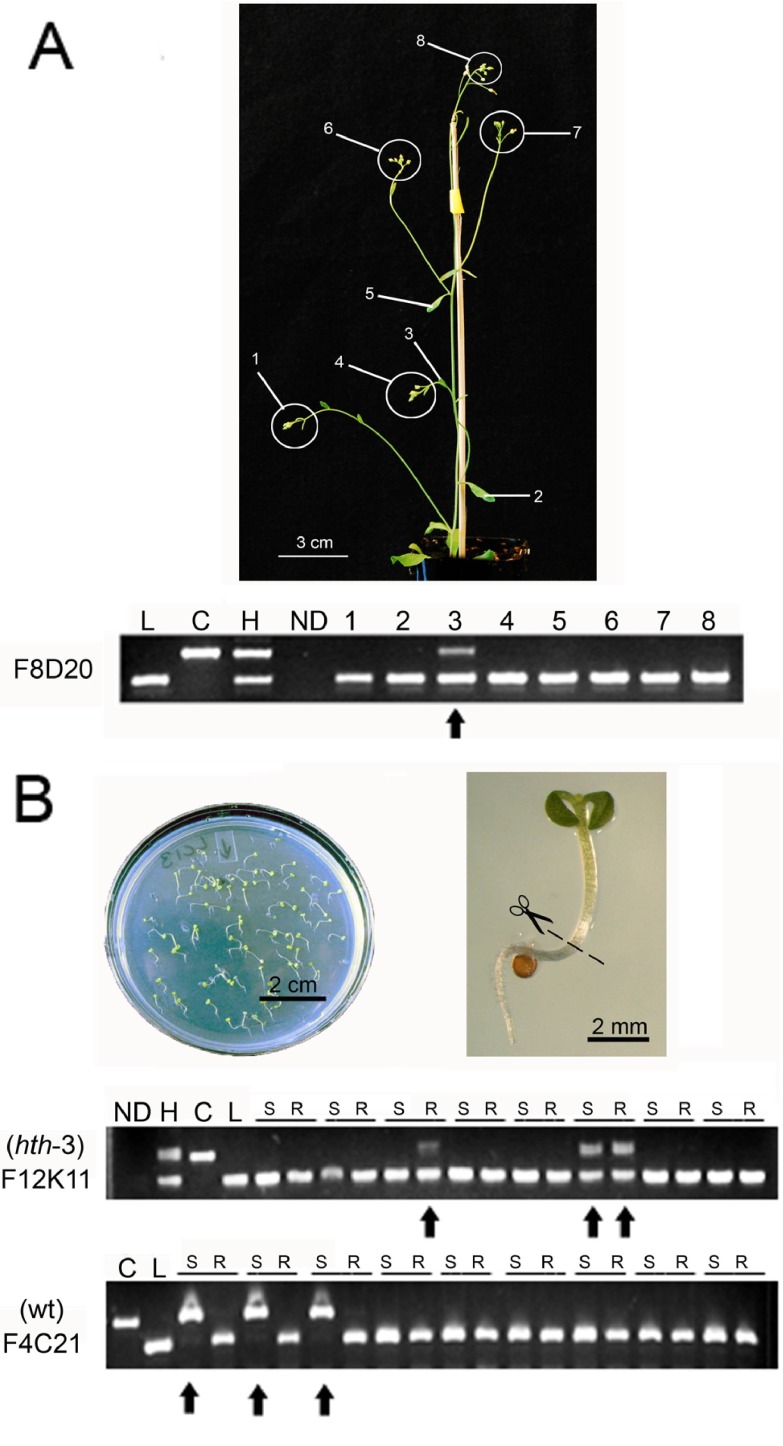
Molecular analysis of an adult mutant plant and bisected mutant and wt seedlings. ( **A**) DNA was extracted from multiple tissue samples and PCR-amplified using F8D20 primers. A novel PCR amplicon product corresponding in size to the insertion allele (C) was detected in *hth-7* tissue sample 3 (arrow). ( **B**) Sterile seeds were sown onto petri plates (top left) and 5-day old seedlings cut at the root-shoot junction (illustrated in the top right panel) and genotyped individually. DNA extracted from shoot (S) and root (R) samples derived from individual *hth-3* or wt seedlings were PCR-amplified using F12K11 and F4C21 primers, respectively. Samples were loaded in pairs (indicated by horizontal bars). Novel amplicon bands were detected in five seedling samples (arrows) that correspond in size to the insertion allele (C). In one *hth-3* sample, both organs (S, R) had a novel band, while a novel amplicon was detected only in the root in a second sample. In three cases, DNA extracted from wt seedlings gave rise to novel bands corresponding in size to the insertion allele (C) (arrows, S). In both cases, the parent plant was homozygous for the deletion allele (L) at the corresponding marker. Heterozygote (H), no DNA control sample (ND).

To test whether the observed genetic discordance between parent and offspring was due to sectoring, multiple tissue samples were collected from individual adult plants and indel marker profiles compared between these different samples. Molecular analyses confirmed that some tissue samples taken from individual *hth* mutant plants had novel marker profiles. For the plant shown in Figure 3A, seven out of eight samples scored homozygous for the Landsberg deletion marker as expected, however, one sample produced two amplicon products, one of which co-migrated with the Landsberg deletion allele while a second larger amplicon co-migrated with Columbia insertion allele.

To test whether sectors could be detected earlier in development, the molecular genotype of shoots and roots of single seedlings grown under sterile conditions were compared to one another. On the assumption that wild type plants would not produce sectors, identical tests were also conducted on wt hybrid lines as negative controls. In the majority of cases, as expected, there was a perfect correspondence between the molecular profiles of root and shoot. However, in some cases, individual seedlings were found to have molecular signatures that differed between the two organ systems (10/44 *hth-3*; 1/50 *hth-4;* 9/76 *hth-7*; Figure 3B). Surprisingly, wt hybrid seedlings also showed novel genotypes when roots and shoots from the same seedling were compared (10/184 wt hybrids; Figure 3B).

### Markers are discordant with parental DNA sequences

**Figure 4.**
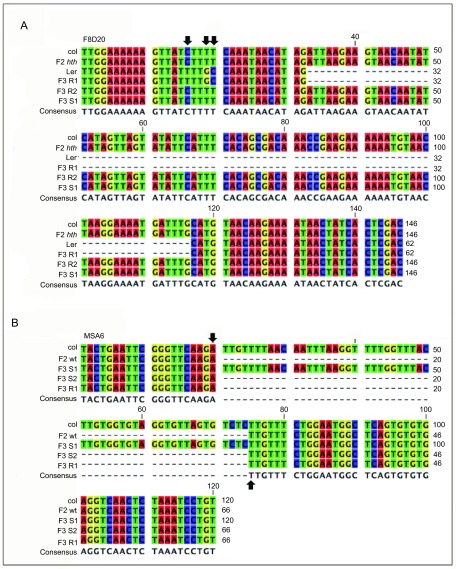
DNA sequence alignments showing F8D20 and MSA6 indel loci. ( **A**) The F2 *hth-3* parent (F2 *hth*) shares sequence identity with 2 of 3 DNA clones isolated from this single *hth-3* seedling (F3 R2 and F3 S1). DNA sequence data obtained from a root clone (F3 R1) shares identity with the Landsberg sequence (Ler), including 3 flanking sequence polymorphisms (arrows) and a corresponding 85 base-pair deletion. The Columbia reference sequence (Col) is shown on the top line of the alignment. ( **B**) The *HTH* wt hybridparent (F2 wt) shares sequence identity with 2 of 3 DNA clones isolated from this single seedling (F3 S2 and F3 R1). DNA sequence data obtained from one shoot clone (F3 S1), however, reveals a 54 base-pair insertion sequence (junctions shown by arrows) and shares identity with the Columbia reference sequence (Col).

A subset of amplicon samples were subjected to DNA sequence analyses in order to determine their molecular features. Sequence analyses of DNA clones derived from individuals where the non-parental amplicon co-migrated with the smaller deletion allele showed identity with the Landsberg deletion marker ( Figure 4). In two instances, polymorphisms immediately upstream of the deletion were also detected ( Figure 4A). As indicated, the Landsberg accession differs from Columbia at these exact three nucleotides. DNA sequence analysis of novel amplicons that co-migrated with the larger insertion allele showed that this seedling shoot had acquired a 54-nucleotide insertion that shares identity with the Columbia reference genome ( Figure 4B). This same insertion was absent in the F2 parent plant. These particular seedlings descended from the same wt hybrid parent plant as the F3 progeny whose profiles are shown in Figure 3B.

### Sectors have complex genotypes

**Figure 5.**
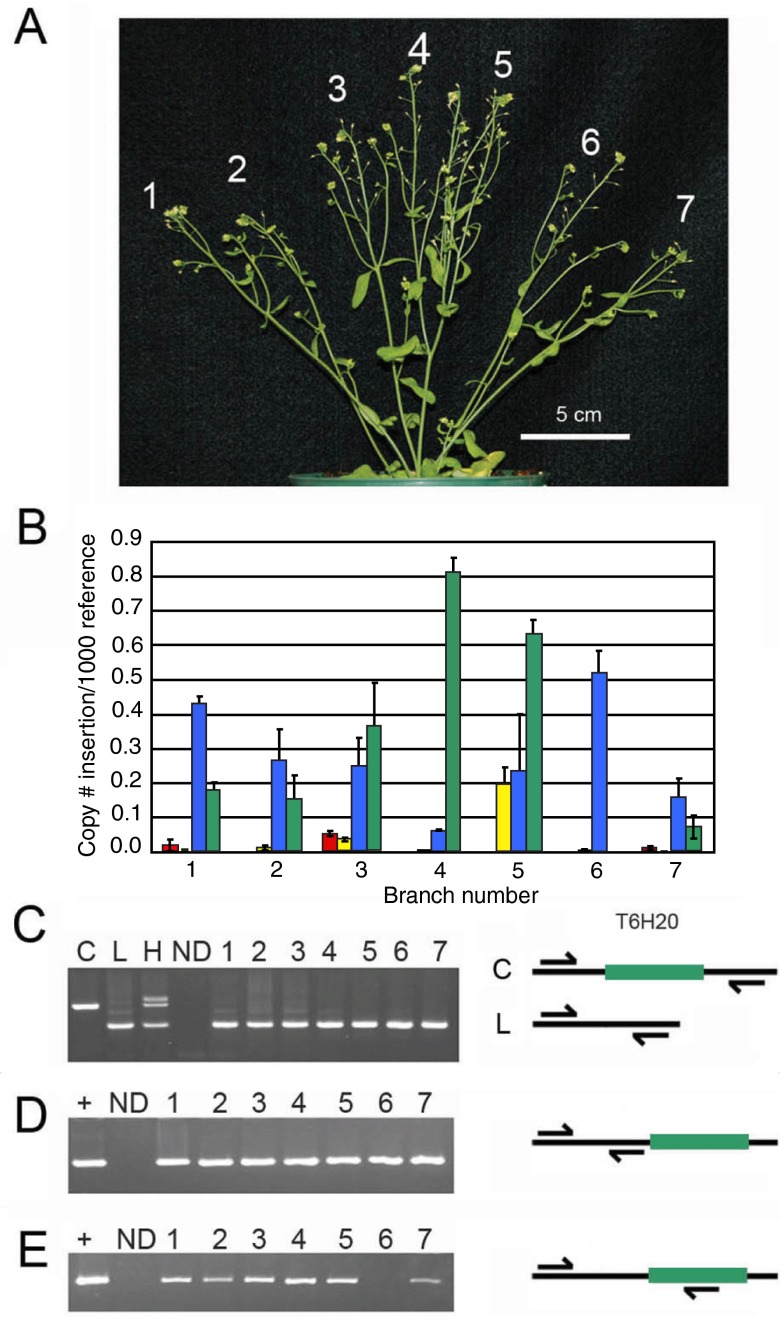
Relative genomic copy number of insertion sequences in a *hth-7* mutant plant. ( **A**) DNA was extracted from branches 1–7 of this *hth-7* mutant plant and amplified using qPCR or standard PCR reactions. ( **B**) Graphical representation of qPCR results using four different indel markers (F8D6 (red), F15H11 (yellow), T14G11 (blue), and T6H20 (green)). Colored bars show the number of insertion sequences per 1000 copies of the reference sequence (lines indicate standard error of the mean, n=3). All 7 samples showed novel insertion sequences. ( **C**) Standard PCR-amplification using T6H20 primers showed amplicons that corresponded exclusively to the deletion allele (L). Primer positions (arrows) relative to the T6H20 indel (green box) are depicted to the right of the gel image. ( **D**) Pooled amplicon product from T6H20 reference primers demonstrate that this region was amplified equally in all samples, as was the positive control (+). The reference sequence is upstream of the T6H20 insertion marker, as depicted on the right. ( **E**) Quantitative PCR using a primer anchored within the T6H20 indel gave rise to amplicons that corresponded in size to the positive control (+). No product was amplified from sample six. T6H20 indel (green box), Columbia (C), Landsberg (L), heterozygote (H), no DNA control sample (ND).

**Figure 6.**
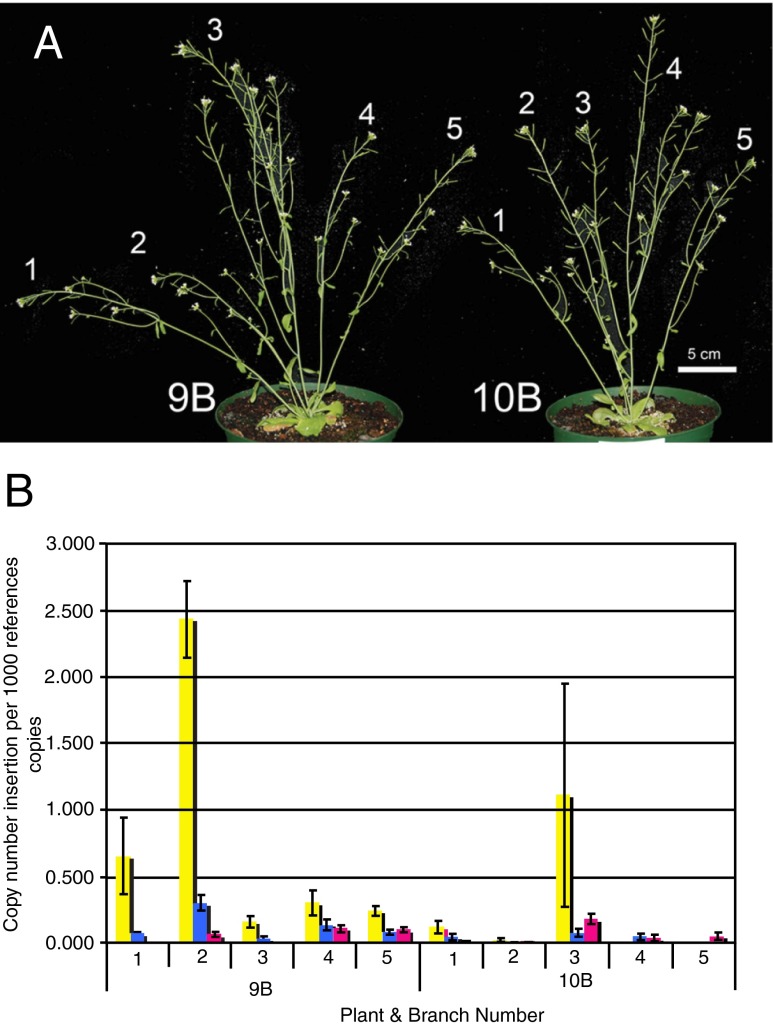
Relative genomic copy number of insertion sequences in two wild type plants. ( **A**) DNA was extracted from branches 1–5 of two wt hybrid plants (9B and 10B) and amplified using qPCR. ( **B**) Graphical representation of qPCR results using three different indel markers ((F15H11 (yellow), T14G11 (blue), and MGI19 (pink)). Colored bars show the number of insertion sequences per 1000 copies of the reference sequence (lines indicate standard error of the mean, n=3). Novel insertion sequences could be detected in all 10 samples.

To obtain an estimate of sector size, tissue samples were subjected to quantitative assays where the copy number of a genomic reference sequence immediately flanking the marker of interest was compared to the copy number of a sequence internal to that particular insertion marker ( Figure 5 and Figure 6). Hybrid plants verified to be homozygous for a deletion at specific indel markers were subjected to the quantitative assays. The quantitative polymerase chain reaction (qPCR) data reveal two remarkable findings. First, the majority of tissue samples collected from individual *hth* mutant plants tested positive for the presence of at least one insertion marker ( Figure 5). In addition, multiple insertion sequences could be detected in many of the tissue samples tested ( Figure 5B). In most instances the copy number of any given insertion sequence, relative to the reference, was very low (less than one copy per 1000). Second, wt hybrid plants also showed evidence of sectors with novel genotypes ( Figure 6). Only two out of four wt plants tested, however, showed evidence of novel insertions.

Colx194E9, F1 #5, plant 54 raw data, F6D8 indel marker. F6D8 indel marker initial data analysis of qPCR results.

Colx194E9, F1 #5, plant 54 raw data, T14G11 indel marker. T14G11 indel marker initial data analysis of qPCR results.

Colx194E9, F1 #5, plant 54 raw data, F15H11 indel marker. F15H11 indel marker initial data analysis of qPCR results.

Colx194E9, F1 #5, plant 54 raw data, T6H20 indel marker. T6H20 indel marker initial data analysis of qPCR results.

Colx194E9, F1 #5, plant 54 raw data, T6H20 indel marker (2). T6H20 indel marker initial data analysis of qPCR results (2).

LC13, wt hybrid plants 9B-10B raw data, T14G11 indel marker. T14G11 indel marker initial data analysis of qPCR results.

LC13, wt hybrid plants 9B-10B raw data, F15H11 indel marker. F15H11 indel marker initial data analysis of qPCR results.

LC13, wt hybrid plants 9B-10B raw data, MGI19 indel marker. MGI19 indel marker initial data analysis of qPCR results.

## Discussion

By employing classical genetic approaches in conjunction with low and high-resolution molecular methods, we show that one Arabidopsis plant can have multiple genotypes. We have found instances of intra-organismal variation in different genetic backgrounds, in plants reared in different growth chambers, at different developmental stages and under sterile growth conditions. Furthermore, the incidence of sectoring and genetic discordance appears to be in some way conditioned by the *hth* mutant background as we found a consistently higher frequency of genetic discordance within single *hth* plants as compared to *HTH* wt plants. This was also true for shoot and root systems compared between aseptically grown seedlings and for tissue samples taken from adult plants and subjected to qPCR. Of critical importance, in showing that single Arabidopsis plants are genetic mosaics, experimental error due to cross-pollination and seed contamination can be completely discounted. To the best of our knowledge, this is the first report that documents the spontaneous but targeted appearance of unique genomic insertions at multiple discreet loci in single plants.

Only two other cases of spontaneous genomic insertions have been reported in plants that similarly could not be explained by any previously known mechanism. In both cases the insertion was non-random and targeted a specific locus. In the case of flax, the insertion sequence was 5.7 kilobase pairs in size ^16^ while in rice the insertion was comparatively small, being only 34 base pairs in size ^33^. Our data suggest that these reported cases of spontaneous genomic insertion events, like the sequence changes reported here, occur by a process intrinsic to the plant. As before, we propose the possibility that Arabidopsis plants harbor a cryptic store of sequence templates that can overwrite the parentally contributed genomes by a template-directed mechanism ^22^.

If intrinsic drivers of genetic variation exist in inbreeding plant species, have additional incidents of cryptic genetic variation been documented in other systems? We believe that in soybean and cauliflower such events have indeed been reported and presented as cases of enigmatic phenotypic variation ^8, 9, 34^. In other studies, molecular data have been featured. Again in flax, for example, molecular assays have demonstrated that heritable phenotypic changes induced by environmental shifts are accompanied by reproducible locus-specific copy number changes in genomic DNA ^16– 18^. In soybean, reproducible non-random changes in restriction length polymorphic markers induced by *in vitro* ^35, 36^ culturing of root explants have also been documented ^19^. Genomic changes manifesting similar hallmarks of biased sequence alterations have also been described in rice ^21, 33^ and corn ^37^ hybrids, as well as in Arabidopsis ^38– 40^.

In long-lived arborescent plants, intra-organism genetic variation has been demonstrated in a variety of systems ^3, 36, 41^. The fitness benefits have also been validated using models that test whether the production of genetically divergent modules is an effective strategy for achieving adaptive co-evolution with organisms that feed on or infect the plant ^35, 42, 43^. Models testing fitness benefits of module-level selection show that this is an effective strategy for achieving adaptive co-evolution between long-lived trees and short-lived herbivores when individual tree branches diverge genetically ^35^. Furthermore, this held true across a range of assumptions, even when reproduction was predominantly asexual. However, the fitness benefits were only fully realized for sufficiently long-lived trees that experienced strong selection ^35^. This fitness paradox is not exclusive to plants but also is relevant to organisms outside of the plant kingdom that have remained evolutionarily robust even though reproduction is predominantly asexual ^43^.

For a short-lived organism such as Arabidopsis, what adaptive value would within-organism genetic variation have? One possibility is that this heterogeneity offsets the predicted decline in genetic variation that should result from inbreeding. Plant development is open-ended and reiterative, allowing for the continuous output of repetitive units or modules that function to support the growth and reproduction of the individual. When combined with developmental plasticity and the absence of a sequestered germ line, modular development may actually drive plants toward becoming genetically heterogeneous ^41, 43– 45^. As posited by Whitham and Slobodchikoff ^3^, somatic sector formation permits the introduction of genetic variants into the gene pool either through vegetative propagation or through sexual reproduction. As these authors point out, germ line cells are derived from somatic tissues that arise late in the developmental history of the plant and therefore somatic mutations are more likely to introduce genetic variation than mutations that arise in the gametes ^3, 4, 46^. By expanding the window of tolerance for genetic variation, plants may be afforded a better adaptive strategy given lifestyle constraints. The versatility of modular development combined with tolerance for genetic variation may allow plants to adapt at rates tailored to pathogen life cycles ^1^ or to relatively expanded time scales, such as those affecting climate change. Even though self-fertilization is thought to have evolved approximately one million years ago ^12^, Arabidopsis plants have not suffered the consequential genetic erosion but have continued to thrive.

In addition to benefiting from a natural tendency toward genetic heterogeneity, the plant genome itself is thought to buffer the cost of having limited genetic diversity. In wild relatives of Arabidopsis the genome is thought to be highly dynamic and to respond to changes in environmental conditions or other extrinsic factors ^42, 47^. Genome responses include elevated rates of homologous recombination that persist for multiple generations ^48^, changes in copy number ^49^ and modulation of epigenetic gene regulation ^50^. Pervasive genetic buffering ^46, 51^ ensures that phenotypes with potentially deleterious consequences are attenuated. In addition to the genome responses listed above, our findings suggest that an intrinsic source of genetic variation can be leveraged to enhance the diversity in genetic output achieved by Arabidopsis plants.

In considering alternate template-dependent mechanisms, such as gene conversion or homologous recombination, none can account for the *de novo* appearance of unique sequence insertions. Nevertheless, it is possible that the insertion or deletion of small DNA sequence tracts, as described here, could reflect the activity of transposable elements ^52, 53^. However, numerous lines of evidence argue against this possibility. For instance, when novel amplicons were detected, they co-migrated with their corresponding insertion or deletion allele and did not show size heterogeneity, as would have been expected for transposon-driven excision or insertion events. Sequence data confirm that deletion events reproducibly eliminate a fixed length of sequence while insertion events reproducibly introduce a fixed sequence tract and both events repeatedly target precise genomic sites. Insertion and deletion events do not appear to produce obvious junction sites with altered nucleotides. Similarly, insertion events introduce sequences that share identity with the Columbia reference genome and do not appear to be chimeric gene or genome fragments. Furthermore, transposable element-mediated events cannot account for the fact that these insertion sequences appear to be generated *de novo* since no comparable conserved region of homology exists elsewhere in the host genome, as demonstrated by our qPCR data. Lastly, as determined by DNA database searches, none of the indel markers used in this study share significant sequence homology with annotated Arabidopsis transposable elements.

If the genome of an intensely studied model organism such as Arabidopsis is subject to modification by the template-directed mechanism we propose, why has this phenomenon not been described previously? Our research shows that target choice and methodological approach are critical in differentiating these genomic events from other processes that also modify DNA sequences. Based on our findings, the only genomic targets that are truly diagnostic of this phenomenon are deletions. To the best of our knowledge, deletions alleles have been used in genetic studies precisely because they are known to be stable and not to revert but have not been used to study phenomena related to epigenetic inheritance. There is no generalized precedent for genetic instability of deletions and assuming otherwise would go against an established biological paradigm. Polymorphic molecular markers such as single nucleotides, simple sequence repeats, or insertions that are subject to alterations by other processes will not provide sufficient resolution to differentiate mechanism, even though they are also likely targets for this process. In particular, our findings may explain why genome sequencing efforts have failed to register these sequence deviations or, if detected, why they may have been attributed to sequencing error and eliminated during curation. One possibility that immediately emerges from this prediction is that raw sequence data contained in existing genome database archives may already contain evidence of extra-genomic sequence information, revealed by features such as highly biased loci-specific "errors".

Collectively, our genetic and molecular data show that many, and perhaps most, insertion events occur somatically in both seedlings and adult plants. Sectoring may therefore be a constitutive process that takes place throughout development but may be limited such that, at any given time, only a few cells host these genetic changes. Importantly, this may explain why sequence changes seen in revertant *hth* progeny have rarely been found to affect both alleles. Although sexual transmission of non-parental markers clearly does occur ^22^, the fact that we have not found *HTH/HTH* progeny among seed-derived offspring suggests that sectors populating the gamete forming lineages are unstable or very rare. The qPCR data are consistent with this supposition. However, it is also possible that mechanistic differences exist between somatic and germ line tissues or that insertion events remain dynamic, limiting sexually transmitted changes to those that stabilize. It is also possible that certain genetic backgrounds condition this process as suggested by the greater number of events detected in *hth* mutants.

In addition to validating our genetic and molecular data, the qPCR results extend those findings and suggest that the genetic make up of individuals can be surprisingly complex. Our data show that each plant can produce multiple discreet sectors, at many different growing points and each with unique marker profiles. This finding implies that sectoring may be a relatively common occurrence, even in wt genetic backgrounds. Since the adult plants used for these experiments were left largely intact and only a small proportion of the plant sampled, many more sectors may have been present than quantified. As such, it is possible that our current census underestimates the frequency with which these smaller islands of genetic variation arise. Although sectors are more readily detected using qPCR, this method cannot distinguish, for example, between copy number variation within a small cluster of cells versus multiple cells that remain strictly diploid and are clonally related. Similarly, it is not possible to distinguish whether one sector hosts the full complement of genetic sequence changes, whether independent events occur in multiple discreet sectors, or if sectors overlap. Visualization of sectors in living tissue or tissue sections should help distinguish between these possibilities.

In addition to models demonstrating the fitness benefits of module-level selection ^35^, computational models provide surprisingly strong support for an ancestrally based "error-correcting" mechanism such as the one we propose to exist in Arabidopsis plants ^54^. In these constrained-optimization simulations, the evolutionary benefit of "genetic repair" strategies was compared between populations that access repair templates derived either from parents, grandparents or great-grandparents. Interestingly, a grandparent- or great grandparent-based genetic repair strategy is strongly favored over parental repair strategies. Furthermore, simulation results show that using a randomly selected template consistently gave superior results to those achieved using templates from the fittest parent or grandparent. From a biological perspective, such a strategy has considerable merit. Retaining a cache of templates derived from grandparental lineages would guarantee greater allele diversity precisely because the reservoir of allele variants would be deeper and allele redundancy would be less likely to occur. Random selection of templates would be the most parsimonious strategy to affect genome repair, again because it would promote diversity across alleles and between individuals. Since only those individuals that survived in previous generations would contribute to these cached templates, represented alleles would be biased to those that have proven robust under a spectrum of selective pressures.

In summary, the research presented here brings to light five striking findings. First, individual Arabidopsis plants are capable of producing somatic sectors during the course of normal vegetative development. Second, those sectors can have distinct and unique marker profiles and can differ in single nucleotide composition, can acquire small DNA insertions or can experience DNA sequence loss. Third, the *de novo* appearance of genomic insertions supports our original contention that cryptic sequence templates drive some of these changes ^22^. Fourth, this phenomenon can be detected in wt genetic backgrounds raising the possibility that many Arabidopsis lab strains may be genetic mosaics. Finally, this process is genome-wide, impacting all 5 chromosomes, whether or not the target loci reside within genes or between genes.

Our data expand on the ideas put forth by Whitham and Slobodchikoff ^3^ and suggest that sector formation, even in a short-lived organism like Arabidopsis, may be a normal part of development and, furthermore, that the formation of sectors serves to capture novel genetic variation, irrespective of the source of that variation. Models testing the benefit of within organism genetic heterogeneity suggest that the average fitness of the population increases if some individuals within that population are genetic mosaics ^35^. As our data show, not all individuals in the populations we tested showed evidence of genetically distinct sectors but for those individuals that did, the number of sectors varied greatly. Our findings raise the possibility that inbreeding plants and, perhaps other organisms that predominantly propagate asexually, may sequester cryptic sources of genetic variation that can be harnessed to promote greater genetic diversity.
