# Supplementary material for: De novo genetic variation revealed in somatic sectors of single Arabidopsis plants
Source: F1000Res. 2013 Jul 30;2:5. Originally published 2013 Jan 10. [Version 2] doi: 10.12688/f1000research.2-5.v2 (PMC3894806; doi:10.12688/f1000research.2-5.v2)
Supplement: Peer review history

# Peer review: Referee response for version 2

**DOI:** 10.5256/f1000research.1962.r3979

**Published:** 2014-03-05

**Authors:** Andy Pereira

**Affiliations:** 1 University of Arkansas, Fayetteville, AR, USA

This new version has adequately addressed my queries.

I have read this submission. I believe that I have an appropriate level of expertise to confirm that it is of an acceptable scientific standard.

# Peer review: Referee response for version 2

**DOI:** 10.5256/f1000research.1962.r1789

**Published:** 2013-09-11

**Authors:** Igor Kovalchuk

**Affiliations:** 1 University of Lethbridge, Lethbridge, AB, Canada

I have no further comments on this paper.

I have read this submission. I believe that I have an appropriate level of expertise to confirm that it is of an acceptable scientific standard.

# Peer review: Referee response for version 2

**DOI:** 10.5256/f1000research.1962.r1650

**Published:** 2013-08-30

**Authors:** David Oppenheimer

**Affiliations:** 1 Department of Botany, University of Florida, Gainesville, FL, USA

The authors have made the changes I suggested for version 1, and I require no further additions to their report.

I have read this submission. I believe that I have an appropriate level of expertise to confirm that it is of an acceptable scientific standard.

# Peer review: Referee response for version 1

**DOI:** 10.5256/f1000research.752.r746

**Published:** 2013-01-31

**Authors:** David Oppenheimer

**Affiliations:** 1 Department of Botany, University of Florida, Gainesville, FL, USA

In the current manuscript, Lolle and colleagues show that plants can spontaneously produce mosaic sectors, and they show that insertion sequences can arise de-novo. Importantly, growing the hth plants in the absence of pollen donors did not eliminate the appearance of HTH plants in the next generation. It is hard to come up with a model for how one could get 1.5% wt plants unless the researchers were covered in wt pollen while tending to the isolated hth plants. It would be useful for the authors to remind the reader that the hth mutants are in the Ler background.

The authors should state the degree to which they tried to eliminate PCR contamination (setting up reactions in UV-sterilized hoods, positive displacement pipets, etc.).

Some important questions/comments:

- What are the allele-specific molecular markers used to genotype the plant shown in Figure 1B? Is the marker used one of the Col/Ler indel markers? What tissues do the individual lanes represent (individual flowers, stem tissue, etc.)? 
- On Page 8, the authors reared the plants in plastic cones, which they conceded does not eliminate outcrossing. Although the frequency of deviation from the expected profile is significantly higher than that due to outcrossing, it would have been better to grow the plants in isolation. This will remain an important criticism, as outcrossing frequencies rely on many variables, and it is possible (albeit difficult) to completely eliminate the possibility of outcrossing.
- On page 8, the authors should clarify the experimental details to make it easy for the readers to follow. The authors should state (if I am following it correctly) that: homozygous Ler hth mutants were crossed with Col wt plants to generate the F1 plants. F1 plants were grown and allowed to set seed. The F2 plants were screened for hth mutants which were saved for F3 analysis. All the markers listed in Figure 2 were used to genotype the individual F2 plants. The individual F2 plants chosen for further study showed the homozygous Ler indel pattern. F3 seedlings were analyzed to address whether or not the marker patterns were stable.
- The key results in Figure 3 cannot be explained by outcrossing, period. 
- Page 8, Change "Markers are discordance…" to "Markers are discordant…"
- Page 9, ‘targeted appearance of unique genomic insertions’: it might be better to say ‘re-appearance of the allele from a previous generation’.

General:

Since HTH has not been cloned yet, this adds an additional layer of complexity to the story. Nonetheless, molecular data were completely absent when transposable elements were discovered, so the identity of HTH is not crucial to the story of de novo genomic changes other than to further understand the nature of the reversion events.

The key aspects of the Peng and Mercier papers were that neither lab was able to find revertants when the plants were grown in isolation. These authors then concluded that there was no non-mendelian mechanism at work, and therefore the rest of the arguments were moot. However, the inability to reproduce the phenomenon does not mean that the phenomenon does not exist; it only means that the Peng and Mercier labs were not able to reproduce the appropriate growth conditions to see the effect. This is not unexpected given that an uncharacterized stress response may be at play here leading to the reversion phenotype.

In my own work with particular *Arabidopsis* mutants that are mostly sterile, we do see revertant sectors, albeit rarely. With one particular mutant, a single inflorescence showed many full seed pods even though the other inflorescences on the same plant showed completely empty pods. Clearly this is not seed or pollen contamination. We have yet to follow up on this rare observation, mainly because it is hard to study rare phenomena.

I have read this submission. I believe that I have an appropriate level of expertise to confirm that it is of an acceptable scientific standard.

# Peer review: Referee response for version 1

**DOI:** 10.5256/f1000research.752.r745

**Published:** 2013-01-31

**Authors:** Igor Kovalchuk

**Affiliations:** 1 University of Lethbridge, Lethbridge, AB, Canada

There is nothing 'wrong' with the paper, although this research is still quite controversial. I would still like to see the mRNA and DNA from *hth* individual plants to be sequenced and compared. Also, if genetic recombination mechanism is in play and if mRNA needs to be converted to cDNA for integration, one could show that some of the mutants involved in recombination or in reverse transcription would be impaired in the process of acquisition of new alleles.

All the experiments were done correctly, but as I said the story is far from completion.

I have read this submission. I believe that I have an appropriate level of expertise to confirm that it is of an acceptable scientific standard.

# Peer review: Referee response for version 1

**DOI:** 10.5256/f1000research.752.r729

**Published:** 2013-01-30

**Authors:** Andy Pereira

**Affiliations:** 1 University of Arkansas, Fayetteville, AR, USA

This paper describes the occurrence of somatic sectors in *hth* mutant genotypes, with the frequency of rare visible sectors at a specific locus being 1/300,000. However, if there is a selective advantage during development, such sectors might be higher and could also produce clonal selected events and easily be identified by PCR.

The data provide adequate support against contamination by outcrossing as a possible explanation of the results.

Minor comment: should change the word 'vernalized' to 'stratified' in the Methods section, sentence beginning "Arabidopsis seeds derived from these stocks..."

I have read this submission. I believe that I have an appropriate level of expertise to confirm that it is of an acceptable scientific standard.
